# Should women under 50 be screened for breast cancer?

**DOI:** 10.1038/sj.bjc.6601966

**Published:** 2004-06-22

**Authors:** S Moss

**Affiliations:** 1 Institute of Cancer Research Cancer Screening, Evaluation Unit Block D, 15 Cotswold Road, Suuton, Surrey SM52 5NG, UK

**Keywords:** breast cancer, screening, mammography

## Abstract

Despite some controversy in recent years, the majority of experts agree on the evidence for effectiveness of breast screening by mammography for women aged 50 years and above, but for those under 50 years, the picture is much less clear. However, the issue remains of importance both to policy makers and to individual women; although the incidence of breast cancer is lower at younger ages, the life years lost due to cancers diagnosed below 50 years amount to a third of all those lost due to the disease.

This article summarises the current position in different countries, and reviews the most recent evidence on effectiveness of screening women below 50 years from randomised trials. It has not been conducted as a systematic review or meta-analysis, of which a number have been performed. It also addresses the range of potential disadvantages of screening with particular reference to this age group.

## CURRENT SCREENING GUIDELINES

The uncertainty is reflected in the variation in current guidelines among countries for screening women below 50 years for breast cancer. A summary of guidelines used in 22 countries, from a survey conducted in 1995 by the International Breast Screening Network (Shapiro *et al*, 1998), reported that while a majority of countries or pilot projects had a lower age limit of 50 years, a number involved a lower limit of 40 years, although in countries such as Sweden and Australia policy can vary by county or state. Japan, which does not use mammography, reported a lower age limit of 30 years. In Sweden, the recommended screening interval is 1.5 years for women aged 40–49 years compared with 2 years for those aged 50 years and above. In the US, the recommendation (updated in February 2002) from the US Preventive Services Task Force is for ‘screening mammography with or without clinical breast examination’ (CBE) every 1 or 2 years for women aged 40 years and above (US Preventive Services Task Force, 2002). The age range was extended below 50 years despite the finding that ‘the strongest evidence of benefit and reduced mortality is among women aged 50–69 years’. The US National Cancer Institute continues to recommend mammography for women in their 40s and older, while the American Cancer Society recommends ‘yearly mammograms’ (plus CBE) starting at 40 years (Smith *et al*, 2003). In 2001, the Canadian Task Force on Preventive Health Care concluded that ‘upon reaching the age of 40 years, Canadian women should be informed of the potential benefits and risks of screening mammography and assisted in deciding at what age they wish to initiate the manoeuvre’ (Ringash, 2001).

The IARC Handbook of Cancer Prevention on breast cancer screening, published in 2002, concluded that the marginal cost-effectiveness of expanding a programme to younger women (aged 40–49 years) greatly depends on its effect on reducing breast cancer mortality as estimated from randomised controlled trials, ‘and that it is likely to be more cost-effective to make other changes’.

## THE EFFECTIVENESS OF SCREENING IN YOUNGER WOMEN

### Evidence from randomised trials

Of the eight randomised trials, which have so far reported mortality results and included women aged 40–49 years at entry, only one (Miller *et al*, 1992) was designed specifically to study the effectiveness of screening in this age group. The remainder included cohorts of women between 40 or 45 and 64 years or older at entry. The relevant trials are summarised in Table 1

**Table 1:** Randomised trials of breast screening

**Study**	**Screening interval (months)**	**CBE included**	**Study**	**Length of follow-up (years)**	**Age range at entry**	**RR (95% CI)**
HIP (Shapiro *et al*, 1988)	12	Yes	1963–1966	18	40–59	0.77 (0.53, 1.11)
Ostergotland (Nystrom *et al*, 2002)	24	No	1978–1981	17	38–49	1.05 (0.64, 1.71)
Kopparberg (Tabar *et al*, 2000)	24	No	1976–1978	17	40–49	0.76 (0.42, 1.40)
Malmo I (Nystrom *et al*, 2002)	18–24	No	1976–1978	19	45–49	0.74 (0.44, 1.25)
Malmo II (Nystrom *et al*, 2002)	18–24	No	1978–1990	12.7	43–49	0.64 (0.45, 0.89)
Gothenburg (Nystrom *et al*, 2002)	18	No	1982–1984	13	39–49	0.58 (0.35, 0.96)
Stockholm (Nystrom *et al*, 2002)	28	No	1981–1983	15	39–49	1.52 (0.80, 2.88)
Edinburgh (Alexander *et al*, 1999)	24	Yes (annual)	1978–1981	14	45–49	0.83 (0.54, 1.27)
NBSS^a^ 1 (Miller *et al*, 2002)	12	Yes	1980–1985	12	40–49	0.97 (0.74, 1.27)

a Mammography+CBE *vs* CBE alone.

RR=relative risk; 95% CI=95% confidence interval.

(the trial in Malmo, which included two cohorts, being included as two separate entries).

There is evidence accumulating from these trials of a possible benefit of screening in women under the age of 50 years. A number of meta-analyses of these trials have been performed (Smart *et al*, 1995). The most recent of these, published in 1997, included an average follow-up time of 12.7 years and estimated a significant 18% reduction in mortality from breast cancer among women aged 40–49 years at entry, invited to screening mammography (RR 0.82, 95% CI 0.71–0.95) (Hendrick *et al*, 1997). An analysis restricted to the five Swedish trials estimated a 29% mortality reduction among women invited to screening (RR 0.71, 95% 0.57–0.89).

The significant benefit seen in this latest meta-analysis is due primarily to updated results from trials in Gothenburg and Malmo. The Gothenburg trial began in 1983/1984, and included approximately 26 000 women aged 39–49 years, of whom 11 724 were randomised to an intervention arm invited for screening by mammography every 18 months. Those in the control arm were invited at the time of the fifth screen of the intervention arm (6–7 years after the date of entry). Owing to the small sample size, a significant reduction was not apparent until 12 years of follow-up, at which time a 45% reduction in mortality from breast cancer was observed in the intervention arm (RR 0.55, 95% CI 0.31–0.96) (Bjurstam *et al*, 1997).

The Malmo trial contained approximately 8000 women under 50 years at entry in the initial cohort randomised in 1977–1978, and a further 17 000 women randomised at age 45–48 years between 1978 and 1990. Pooling the two cohorts, there was a statistically significant reduction in breast cancer mortality in the intervention arm at 12 years of follow-up (RR 0.64, 95% CI 0.45–0.89) (Andersson and Janzon, 1997).

An updated overview of the five Swedish studies, published in 2002, did not include the Kopparberg part of the Two-County trial, but did include a continuation of the Malmo trial (Nystrom *et al*, 2002). In this overview, the median follow-up time was 15.8 years; there was a 20% reduction in women aged 40–49 years at entry (RR 0.80, 95% CI 0.63–1.01), and an analysis by 5 year age groups found no significant heterogeneity.

The Canadian National Breast Screening Study 1 was designed to evaluate the efficacy of the combination of annual mammography, physical examination and the teaching of breast self-examination in reducing the rate of death from breast cancer among women aged 40–49 years at entry. Between 1980 and 1985, the trial randomised 50 430 women aged 40–49 years to an arm offered annual mammography and physical examination (MP), or to an arm offered usual care (UC) after an initial physical examination. After a mean of 8.5 years of follow-up, there was a nonsignificant 3.6% increase in breast cancer mortality in the MP group (RR 1.36, 95% CI 0.84–2.21); at a mean of 13 years follow-up the rate ratio was 0.97 (95% CI 0.74–1.27). An adjustment taking account of mammography done outside the study yielded a rate ratio of 1.06 (95% CI 0.80–1.40) (Miller *et al*, 2002).

This trial made use of a volunteer population, which may not be representative of the general population, and there has been debate over the quality of mammography, and over the effect of the initial examination and education in the control arm. A major drawback of the study has been the lack of statistical power, in part due to lower than anticipated mortality in the control arm, with the result that even after 13 years the confidence intervals are wide (Kopans *et al*, 1994).

### Interpretation of results

The interpretation of the various trial results has been widely debated. Differences between the stage or size distribution of cancers in the control arms have been cited as explaining observed differences in benefit between trials (Narod, 1997), but despite differences in study design there is no statistically significant heterogeneity (Hendrick *et al*, 1997). Since the effect of screening on mortality from breast cancer takes a number of years to become apparent, and all the trials have invited women for routine rescreening at intervals ranging from 18 to 33 months, it is difficult to separate the effect of screening examinations, which took place above and below 50 years. Analyses by age at diagnosis are complicated by the effect of lead-time, the length of time by which diagnosis has been advanced for screen-detected cancers which will lower the age at diagnosis.

The MISCAN simulation model has been used to estimate that 70% of the mortality reduction observed in the Swedish trials in women aged 40–49 years at entry was a result of screening these women after they reached 50 years (de Koning *et al*, 1995), based on a comparison of predicted with observed results. However, the alternative assumption that screening under 50 years has the same effect as that in older women could not be excluded on the basis of this analysis.

This analysis was based on the observed breast cancer mortality reduction of 10% in women aged 40–49 years reported at that time; with longer follow-up, the observed reduction is larger, and further analyses are being conducted in order to update these estimates (de Koning, personal communication).

Reasons for a possible lesser effect of screening in younger women include lower sensitivity of mammography due to a higher prevalence of dense tissue, and a tendency for tumours in young women to be faster growing, implying a need for more frequent screening (Tabar *et al*, 1995).

The results of a meta-analysis (which also included one case–control study) suggested that the summary relative risk of mortality from breast cancer in women aged 40–49 years invited for screening compared with those who were not was lower for studies using two view mammography, and for studies with longer follow-up (Kerlikowske *et al*, 1995).

It appears likely that there will be a benefit in terms of reduced mortality from breast cancer associated with screening below 50 years, and that frequent (e.g. probably annual) screening will be necessary to achieve this. However, the size of the benefit and the age(s) at which screening should be performed are as yet unknown. It is likely that any changes in the effectiveness of screening with increasing age occur gradually, and the choice of age 50 years for subgroup analyses is arbitrary (Kopans, 1998), although this could be viewed as a surrogate for age at menopause.

### Research in progress

A trial in progress in the UK has been designed specifically to address the question of whether offering screening by mammography from 40 years is effective in reducing mortality from breast cancer, compared with the current national policy of inviting women from the age of 50 years (Moss, 1999). A total of 160 000 women aged 40–41 years have been randomised in the ratio 2 : 1 to a control arm or an intervention arm. Women in the latter are offered annual mammography, until 48 years by two views at first screen, and single view at subsequent screens unless indicated otherwise. Women in the control arm receive usual medical care, and those in both arms will receive their first invitation to screening as part of the national programme between 50 and 52 years. The trial began in 1991 and includes 23 centres, all of which are existing NHS breast screening units. The trial is powered to detect a 20% reduction in breast cancer mortality at 10 years of follow-up. Meanwhile, surrogate outcome measures in terms of pathology characteristics are being considered (Anderson *et al*, 2000).

## COSTS OF SCREENING

The financial costs of mammography include those of screening mammography, and further assessment and treatment costs, as well as organisational costs for organised programmes. Screening will also impact on treatment costs. The frequency of screening will affect the cost across a given age range, and most comparisons of cost-effectiveness have assumed a shorter screening interval (12–18 months) below 50 years. However, the results of such comparisons will vary according to the estimate of effectiveness used. De Haes *et al* (1991) concluded that it would be more cost-effective to shorten the screening interval for women aged 50–70 years than to include 2 yearly screening for women aged 40–49 years, based on the 8% mortality reduction then observed in the Swedish Two Counties Study in women under 50 years. An analysis in 1995 by Lindfors and Rosenquist (1995), using a baseline estimate of mortality reduction for women aged 40–49 years of 4% with biennial and 23% with annual mammography, found that including women aged 40–49 years increased the marginal cost per life year saved, but by less than some alternative strategies. Salzmann *et al* (1997) assumed a 16% reduction in breast cancer mortality starting at 50 years for women who begin screening at 40 years and found that the cost-effectiveness of mammography in women aged 40–49 years was about five times that in older women.

## ADVERSE EFFECTS OF SCREENING

In common with all screening tests, there are risks or disadvantages associated with mammography, which need to be weighed against any beneficial effect. Information on the adverse effects of screening is essential to enable decisions to be reached by policy makers on whether screening should be offered, and by women on an individual basis on whether or not to attend.

### False-positive results

Referral for further assessment of women who are subsequently found to be free of cancer is one disadvantage of screening, which will have a cost in terms of resources (Lidbrink *et al*, 1996), and to the women referred. While attempts can be made to keep referral rates low, there is a ‘trade off’ between specificity and sensitivity, since if referral rates are too low, then the cancer detection rates may also be low. Within the NHS breast screening programme, referral rates tend to decrease with age, while cancer detection rates increase in line with underlying incidence, with the result that the positive predictive value of referral for assessment (i.e. the probability that a woman referred will be found to have breast cancer) is likely to be lower below 50 years. The cumulative risk of a false-positive test (in women aged 40–69 years) has been estimated in one US study to be 49% after 10 mammograms (Elmore *et al*, 1998).

A number of studies have looked at the psychological effects of the screening process on women, both for those screened as normal and those referred for further assessment. Routine attendance for screening in general seems to have little effect, although some subgroups experience distress or increased anxiety (Walker *et al*, 1994). The most significant effects are observed in women referred for further assessment with false-positive results. While most studies have found increased anxiety in those women to be fairly short-lived (Cockburn *et al*, 1994; Ellman and Thomas, 1995; Lampic *et al*, 2001), some have reported long-term effects (Lerman *et al*, 1991). However a false-positive result does not appear to deter women from subsequent attendance for screening (Lerman *et al*, 1991; Lightfoot *et al*, 1994). Anxiety is likely to be minimised by the provision of appropriate information and by reducing the time between initial screen and assessment. Timely reporting of results may also reduce anxiety of the effects associated with earlier stages in the screening process.

### Exposure to radiation

The possible harmful effect of radiation resulting from mammography in this age group has been widely debated (Law, 1995; Feig and Hendrick, 1997; Young, 2001). Estimates of breast cancer risk from low-dose radiation exposure are, by necessity, extrapolated from studies of populations exposed to much higher doses, for example atomic bomb survivors, or those receiving medical radiation treatment, and these studies have suggested a greater effect at lower ages of exposure. The estimated risk associated with radiation will depend on whether a linear or quadratic dose response model is chosen, and on whether an additive or relative risk model is used. The average radiation dose for mammography has reduced considerably over the past 15–20 years, and most recent studies have estimated that the benefit of screening is likely to outweigh the risk even in younger women (Law and Faulkner, 2001). Estimates of the average received dose in the UK Age trial are of 2.5 mGy per oblique film and 2.0 mGy per craniocaudal film (Young, 2001); in this study, age was not found to be a significant factor affecting the dose to screened women aged above or below 50 years. A small proportion of women will receive higher doses for reasons such as large breasts requiring more than one film per view.

### Overdiagnosis

Another concern about screening is the possibility of over diagnosis, by the detection of lesions which would otherwise not have presented clinically during a woman's lifetime. In the case of breast screening, there is particular uncertainty over the natural history of ductal carcinoma *in situ* (DCIS), which is infrequently diagnosed in the absence of screening. In the Swedish Two Counties randomised controlled trial, there was most evidence of over diagnosis in women below 50 years (Tabar *et al*, 1992); however, in that trial, the rate of detection of DCIS was comparatively low. In the NHS breast screening programme, 4% of cancers detected by screening in 2000/2001 in women aged above 50 years were *in situ* (NHS Breast Screening Programme Annual Review, 2002). The benefit of detecting and treating DCIS remains an area of debate. A study in the UK found that the majority of DCIS detected by screening was high-grade (Evans *et al*, 2001), and will, therefore, have a high probability of progression to invasive cancer, but others have argued that progression rates may be much lower (Jatoi and Baum, 1995; Baum, 1996). The IARC handbook concludes that studies are required on the natural history of DCIS, and on the impact of detection and treatment of DCIS on the incidence of invasive cancers.

### Screening in high-risk women

Of particular concern in many settings is the management of women at increased risk of breast cancer due to family history of the disease. It is estimated that such cases may account for between 5 and 10% of all cases (Eeles, 1999), and such women will be at increased risk from a young age. It has been estimated that most of the excess risk of familial breast cancer occurs before 50 years (Houlston *et al*, 1992) and it is generally recommended that screening start 5–10 years before the earliest age at diagnosis of affected family members.

Clearly if screening is effective in this age group, then the absolute benefit in this subgroup will be greater, as will the cost-effectiveness. Nevertheless, there is no more evidence for effectiveness in high-risk women than in the general population.

In the UK, two studies are in progress to assess the effect of screening in high-risk women. A randomised controlled trial is comparing the sensitivity of mammography and magnetic resonance imaging (MRI) in high-risk women identified from genetics clinics (The UK MRI Breast Screening Study Advisory Group, 2000). Magnetic resonance imaging does not involve the use of ionising radiation, and hence may be of particular relevance for screening at young ages. However, studies to date have suggested high sensitivity but possibly low specificity. The cost of the technique at present is such that its use is only under consideration for women at high risk. A second study is evaluating mammographic surveillance in women under 50 years with a family history of breast cancer, with the aim of estimating the difference in breast cancer mortality in screened compared with unscreened women, and estimating the cost-effectiveness of regular mammography in this group (MacKay *et al*, 2001).

Ultrasound has also been considered as a screening modality for high-risk women, although there are doubts about both sensitivity and specificity (Teh and Wilson, 1998). One recent study concluded that MRI is likely to be superior as a screening method for such women (Warner *et al*, 2001).

The increased radiation risk in these women will be affected by the choice of model (additive or relative risk). It is possible that some women genetically predisposed to breast cancer may also have increased sensitivity to radiation, and if screened frequently from a young age will also have increased total exposure. An example is ataxia telangiectasia (AT), an inherited autosomal recessive disease. Women who are AT gene carriers are also at increased risk of developing breast cancer; however, there remains debate as to whether this risk is increased by exposure to ionising radiation (Swift *et al*, 1991; Wagner, 1992).

## CONCLUSIONS

Until more information is available, it is difficult to inform either policy makers (in terms of potential changes to national screening programmes) or individual women on the balance between costs and effectiveness of screening below 50 years. It has recently been suggested, on the basis of age-specific incidence rates, that screening in the UK should begin at 47 years (Sasieni and Cuzick, 2003), but the effect of such a policy in terms of mortality reduction is not clear. For example, a number of the randomised trials have observed a lesser effect in women aged 50–54 years at entry.

For individual women, full information on the possible benefits and harmful effects of screening are essential to enable an ‘informed choice’ to be made and the need for accurate and understandable information has been emphasised recently (Baines, 2003; Thornton *et al*, 2003). Only once further information on the effectiveness of screening below 50 years is available, can the cost-effectiveness of any change in policy can be estimated. Decisions on national policy will then depend on comparing cost-effectiveness with other policy options.
